# Administration of *N*-acetylcysteine Plus Acetylsalicylic Acid Markedly Inhibits Nicotine Reinstatement Following Chronic Oral Nicotine Intake in Female Rats

**DOI:** 10.3389/fnbeh.2020.617418

**Published:** 2021-02-03

**Authors:** María Elena Quintanilla, Paola Morales, Fernando Ezquer, Marcelo Ezquer, Mario Herrera-Marschitz, Yedy Israel

**Affiliations:** ^1^Molecular and Clinical Pharmacology Program, Institute of Biomedical Sciences, Faculty of Medicine, University of Chile, Santiago, Chile; ^2^Department of Neuroscience, Faculty of Medicine, University of Chile, Santiago, Chile; ^3^Centro de Medicina Regenerativa, Facultad de Medicina Clínica Alemana, Universidad del Desarrollo, Santiago, Chile

**Keywords:** acetylsalicylic acid, *N*-acetylcysteine, nicotine, oxidative stress, reinstatement

## Abstract

**Background:**

Nicotine is the major addictive component of cigarette smoke and the prime culprit of the failure to quit smoking. Common elements perpetuating the use of addictive drugs are (i) cues associated with the setting in which drug was used and (ii) relapse/reinstatement mediated by an increased glutamatergic tone (iii) associated with drug-induced neuroinflammation and oxidative stress.

**Aims:**

The present study assessed the effect of the coadministration of the antioxidant *N*-acetylcysteine (NAC) plus the anti-inflammatory acetylsalicylic acid (ASA) on oral nicotine reinstatement intake following a post-deprivation re-access in female rats that had chronically and voluntarily consumed a nicotine solution orally. The nicotine-induced oxidative stress and neuroinflammation in the hippocampus and its effects on the glutamate transporters GLT-1 and XCT mRNA levels in prefrontal cortex were also analyzed.

**Results:**

The oral coadministration of NAC (40 mg/kg/day) and ASA (15 mg/kg/day) inhibited by 85% of the oral nicotine reinstatement intake compared to control (vehicle), showing an additive effect of both drugs. Acetylsalicylic acid and *N*-acetylcysteine normalized hippocampal oxidative stress and blunted the hippocampal neuroinflammation observed upon oral nicotine reinstatement. Nicotine downregulated GLT-1 and xCT gene expression in the prefrontal cortex, an effect reversed by *N*-acetylcysteine, while acetylsalicylic acid reversed the nicotine-induced downregulation of GLT-1 gene expression. The inhibitory effect of *N*-acetylcysteine on chronic nicotine intake was blocked by the administration of sulfasalazine, an inhibitor of the xCT transporter.

**Conclusion:**

Nicotine reinstatement, following post-deprivation of chronic oral nicotine intake, downregulates the mRNA levels of GLT-1 and xCT transporters, an effect reversed by the coadministration of *N*-acetylcysteine and acetylsalicylic acid, leading to a marked inhibition of nicotine intake. The combination of these drugs may constitute a valuable adjunct in the treatment of nicotine-dependent behaviors.

## Introduction

Nicotine is a neuroactive alkaloid responsible for the development and maintenance of tobacco addiction (Stolerman and Jarvis, 1995; Pontieri et al., 1996; Merlo Pich et al., 1999). While tobacco is mostly smoked, oral intake of nicotine is well recognized. Tobacco chewing (Gajalakshmi and Kanimozhi, 2015) and nicotine gums (Kostygina et al., 2016) have become public health concerns in Asia and the United States.

Globally, 20% of the population 15+ years of age are current smokers (WHO, 2019). Current smoking cessation pharmacotherapies have largely targeted the nicotinic acetylcholine receptors (nAChRs), as the activation of these receptors mediates the early rewarding effects of tobacco (Coe et al., 2005; Rollema et al., 2007; Polosa and Benowitz, 2011). However, the molecular mechanisms underlying the vulnerability to smoking relapse are multifaceted and the risk of relapse persists even in individuals receiving replacement therapy (Medioni et al., 2005; Etter and Stapleton, 2006).

Recent studies suggest a likely role of astrocytes and microglia in nicotine addiction. Cue-induced reinstatement of nicotine self-administration and chronic nicotine exposure in rats is associated with increased tumor necrosis factor-alpha (TNFα) levels in the prefrontal cortex and nucleus accumbens (Royal et al., 2018; Namba et al., 2020). Consistent with the above, studies in the authors’ laboratory showed that both chronic nicotine intake and nicotine-conditioned place preference were associated with changes in markers of glial inflammation, including an increased length and thickness of astrocytic processes and increased microglial density (cell number/area) in the rat hippocampus (Quintanilla et al., 2018, 2019). Recent studies in mice also showed that chronic nicotine administration and nicotine withdrawal symptoms were associated with changes in microglial morphology and microglial-mediated release of pro-inflammatory cytokines: TNF-α, and IL-1β in striatal tissues (Adeluyi et al., 2019; Saravia et al., 2019). Adeluyi et al. (2019) also showed that microglial activation was associated with increases in reactive oxygen species (ROS) levels in the nucleus accumbens.

Nicotine exposure has also been shown to induce oxidative stress in brain regions responsible for learning and memory in rodents (Bhagwat et al., 1998; Gumustekin et al., 2003; Toledano et al., 2010; Budzynska et al., 2013; Biala et al., 2018). Nicotine induces oxidative stress through several mechanisms. *Per se*, dopamine released by nicotine during the stimulation of the mesolimbic dopamine system is deaminated by monoamine oxidase, generating hydrogen peroxide (Cadet and Brannock, 1998). In addition, dopamine released into the extracellular pH is rapidly auto-oxidized to superoxide radicals and hydrogen peroxide (Cunha-Oliveira et al., 2013). The above findings indicate (a) a nicotine-induced activation of microglia as associated with ROS production and (b) TNF-α increases, known to activate NADPH oxidase (Kim et al., 2007; Basuroy et al., 2009) and generate hydrogen peroxide and mitochondrial superoxide ions (Kastl et al., 2014). Combined, these studies suggest that oxidative stress and neuroinflammation are self-perpetuated in a vicious-like cycle, making relapse a protracted event (Berrios-Carcamo et al., 2020).

The hippocampus appears as the most relevant region to study the induction of brain oxidative stress and neuroinflammation by nicotine given that large increases in ROSs and increases of TNF-α and IL-1β were shown in the hippocampus following chronic nicotine administration to rats (Toledano et al., 2010; Motaghinejad et al., 2020). Recently, Bade et al. (2017) using non-invasive imaging techniques monitored the nicotine-induced activation of specific subregions of the rat brain. They reported that of all central nervous system reward regions, the hippocampus was the one most activated by the 1-week nicotine infusion, while resonance imaging in the nucleus accumbens, amygdala, and prefrontal cortex were less affected.

Oxidative stress (and associated neuroinflammation), as induced by chronic nicotine intake, inhibits astrocyte glutamate transporters that regulate glutamate homeostasis (Berrios-Carcamo et al., 2020). Previous research has established that alterations of glutamate homeostasis in the prefrontal cortex and nucleus accumbens contribute to the reinstatement of drug-seeking behavior (Kalivas, 2009). For several drugs, including cocaine, ethanol, and heroin, it has been shown that reinstatement of drug seeking by conditioned cues is associated with glutamate overflow in the nucleus accumbens (Baker et al., 2003; LaLumiere and Kalivas, 2008; Gass et al., 2011). For nicotine, this glutamate overflow at the tripartite synapse was associated with a reduced ability to lower glutamate levels from the synaptic cleft, due to reduced levels of the glial glutamate transporter GLT-1 in the nucleus accumbens and striatum (Knackstedt et al., 2009; Gipson et al., 2013; Alasmari et al., 2017). Further, nicotine self-administration decreased the levels of the xCT cystine/glutamate transporter, in the hippocampus and nucleus accumbens in rats (Knackstedt et al., 2009; Alasmari et al., 2017). The xCT transporter exchanges extracellular cystine for intracellular glutamate and normally is the main source of extracellular glutamate (McBean, 2002).

The prefrontal cortex is an important brain region since upon addictive drug seeking and reinstatement there is a large increase in both prefrontal firing activity and glutamate release in the nucleus accumbens derived from prefrontal afferents neurons (Kalivas, 2009). It is noted that the nucleus accumbens is primarily a relay from the hippocampus and prefrontal glutamatergic structures onto medium spiny neurons (Britt et al., 2012; Scofield and Kalivas, 2014).

While the prefrontal cortex is an important region in the glutamatergic decision-making system, little is known about the effects of nicotine exposure on the expression of the glutamate transporter-1 (GLT-1) and xCT in the prefrontal cortex. Two previous sub-chronic studies did not reveal changes in GLT-1 or xCT in the prefrontal cortex of rats receiving nicotine via self-administration or via minipumps for 21 days (Knackstedt et al., 2009) or in mice exposed phasically (not continuously) to e-cigarette vapor containing nicotine (Alasmari et al., 2017). However, no studies have assessed whether changes occur in GLT-1 or xCT in the prefrontal cortex of rats that have consumed nicotine continuously for long periods (equivalent to years for chronic smokers), an area addressed in the present work.

The likely mechanism of inhibition of the two glutamate transporters GLT-1 and xCT is an oxidation of their cysteine residues (Trotti et al., 1997, 1998; Berrios-Carcamo et al., 2020). Additionally, TNFα leads to the downregulation of GLT-1 gene expression (Szymocha et al., 2000; Wang et al., 2003; Sitcheran et al., 2005). Noteworthily, a dysfunction of either one of the two transporters increases postsynaptic glutamate tone, namely, (i) a dysfunctional GLT-1 prevents the glial glutamate removal at the tripartite synapse while (ii) a dysfunctional xCT transporter, being unable to activate glutamate efflux, reduces the inhibition of the inhibitory mGlu2/3R (Xi et al., 2002). Both effects lead to higher glutamate levels at the tripartite synapse, primarily during cued and drug-induced reinstatement, thus driving a drug-seeking behavior (Moran et al., 2005).

The above findings suggest that both antioxidants and anti-inflammatory agents, via the restoration of normal glutamate homeostasis, would reduce the cue-induced reinstatement of nicotine-seeking behavior. *N*-acetylcysteine, an antioxidant and a prodrug in the generation of cysteine, has been reported to inhibit cue-induced nicotine seeking, an effect that was accompanied by normalization of the reduced GLT-1 expression induced by nicotine self-administration in the nucleus accumbens (Namba et al., 2020). The finding that *N*-acetylcysteine also activates the xCT cystine/glutamate transporter (Baker et al., 2003; Knackstedt et al., 2009; Alasmari et al., 2017) suggests that *N*-acetylcysteine could normalize the nicotine-induced overflow of glutamate seen in the nucleus accumbens during cue-induced nicotine reinstatement (Gipson et al., 2013), both by increasing the xCT cystine-glutamate transporter levels and importantly by increasing cystine levels a substrate for the antiporter, thus activating the inhibitory presynaptic metabotropic mGlu2/3 receptor.

In line with the existence of a self-perpetuating oxidative stress–neuroinflammation relationship, it was recently reported that the combined administration of the antioxidant *N*-acetylcysteine plus the anti-inflammatory acetylsalicylic acid inhibited chronic alcohol intake and alcohol reinstatement to a greater extent than that induced by each agent alone (Israel et al., 2019). A number of studies have shown that aspirin activates the synthesis of the peroxisome proliferator-activated receptor gamma (PPAR-γ) known to have both anti-inflammatory effects (Jiang et al., 1998; Ricote et al., 1999; Pascual et al., 2007) and to activate brain GLT-1 transcription, increasing GLT-1 protein levels (Romera et al., 2007). Six PPAR response elements (PPREs) exist in the GLT-1 gene (Romera et al., 2007). Indeed, acetylsalicylic acid was found to markedly increase the levels of GLT-1 in the prefrontal cortex (Israel et al., 2019).

Given the above, we hypothesize that should a prolonged nicotine exposure result in oxidative stress and neuroinflammation in the hippocampus and a reduction of GLT-1 and xCT in the prefrontal cortex, the coadministration of acetylsalicylic acid that increases GLT-1, added to the effect of *N*-acetylcysteine which increases the levels and activity of the cystine glutamate transporter xCT, would provide a greater inhibitory effect on the reinstatement of oral post-deprivation nicotine intake, than that induced by *N*-acetylcysteine and acetylsalicylic acid administered separately. To further test the hypothesis, we analyzed if the coadministration effects of both drugs were accompanied by increases in prefrontal cortex mRNA levels of GLT-1 and xCT. Finally, we evaluated whether blocking the xCT cystine/glutamate antiporter by the inhibitor sulfasalazine blunted the inhibition of oral nicotine intake exerted by *N*-acetylcysteine.

## Materials and Methods

Two-month-old UChB female rats (Quintanilla et al., 2006) were used in the experiments. The rationale for using female rats in this study is that female rats show a higher nicotine-seeking behavior compared to males (Donny et al., 2000; Torres et al., 2009). Additionally, females maintain stable body weights (within 10%) over time—of value in long-term studies. Animals were maintained on a 12-h light/dark cycle (lights off at 7:00 PM) and were regularly fed with a soy protein, peanut meal rodent diet (Cisternas, Santiago, Chile). Given that rats do not readily voluntarily consume nicotine orally, the animals were pretreated with an intraperitoneal dose of nicotine (0.6 mg/kg/day), for 2 weeks, which promoted a subsequent escalation of oral nicotine intake voluntarily when offered the choice of water and nicotine solution using a two-bottle paradigm (Quintanilla et al., 2018, 2019). Details of this procedure are described below. All animal procedures were approved by the Committee for Experiments with Laboratory Animals at the Medical Faculty of the University of Chile (Protocol CBA# 0994 FMUCH).

### Drugs

(−)-Nicotine hydrogen tartrate was obtained from Sigma-Aldrich, St. Louis, MO, United States. The nicotine solution for the initial intraperitoneal administration was prepared by dissolving nicotine hydrogen tartrate in saline and adjusted to pH 7.2–7.4 with NaOH (0.1 N) and injected in a volume of 5.0 mL/kg/day, at a dose of 0.6 mg/kg/day (Bashiri et al., 2016) by the intraperitoneal route (Al Shoyaib et al., 2019). The intraperitoneal administration was carried out by holding the rat in supine position with its head tilted lower than the posterior part of the body, and the needle was inserted in the lower right quadrant of the abdomen (at ∼10° angle) with care to avoid accidental penetration of the viscera (Al Shoyaib et al., 2019). The nicotine solution concentrations for oral intake, calculated as free base, were 5, 10, 30, 50, or 60 mg/L (w/v) and were prepared by dissolving nicotine hydrogen tartrate in distilled water every day. *N*-acetylcysteine (NAC) (Sigma, St. Louis, MO, United States) was dissolved in water, adjusted with NaOH to pH 7.2, and administered in a volume of 5.0 mL/kg/day at a dose of 40 mg/kg/day, using the intragastric route of administration by gavage (Conybeare and Leslie, 1988). Briefly, the intragastric administration by oral gavage was carried out using a steel curved needle 70 mm long, 5-gauge, with a tip “bulbed” of 4 mm. For the oral administration the rat was held firmly by the skin of the neck and back so that the head is kept immobile and in line with the back. The needle attached to a syringe was passed into the mouth and after locating the entry to the esophagus is pushed gently into the stomach to initiate the discharge (Conybeare and Leslie, 1988). Acetylsalicylic acid (ASA) (Sigma-Aldrich, St. Louis, MO, United States), was dissolved in water and adjusted with NaOH to pH 7.2 and administered in a volume of 5.0 mL/kg/day at a dose of 15 mg/kg/day, by the intragastric route using oral gavage, as indicated above. This ASA dose is considerably lower than that used as chronic treatment for arthritis in humans (Colebatch et al., 2012). Doses of ASA of 15–30 mg/kg were shown not to generate gastric irritation in rats (Wallace et al., 2004). When the combination of NAC + ASA was administered, both drugs were dissolved in water, adjusted with NaOH to pH 7.2, and delivered in a volume of 5 mL/kg/day by oral gavage (Israel et al., 2019). Sulfasalazine, an xCT transporter inhibitor, was administered intraperitoneally (Al Shoyaib et al., 2019) to achieve a better bioavailability (Bernabucci et al., 2012). Sulfasalazine stock solution was prepared dissolving sulfasalazine in DMSO (80 mg/ml), as previously reported (Bernabucci et al., 2012). Fresh sulfasalazine (referred as SZ) was prepared every day by dilution of the stock solution in isotonic saline and administered intraperitoneally at a dose of 8 mg/kg/day delivered as 5 ml/kg/day (Bernabucci et al., 2012). As reviewed earlier, this dose of sulfasalazine does not have an anti-inflammatory effect (Quintanilla et al., 2020).

### Brain Tissue Samples

We selected the hippocampus to study oxidative stress and neuroinflammation produced by nicotine since, as indicated above, it has been shown that nicotine induces both (i) a greater production of oxidative stress in the hippocampus than in other regions and (ii) an increase of TNF−α and IL-1β in this region (Toledano et al., 2010; Motaghinejad et al., 2020). In addition, the hippocampus is an area involved in the associations of nicotine’s rewarding effects with specific contextual cues (Dols et al., 2000). We dissected the whole left hippocampus for GSSG/GSH assays, while the right brain hemisphere was fixed in 4% paraformaldehyde in phosphate-buffered saline (0.1 M PBS) to obtain coronal sections of the hippocampus (20 μm thick) to evaluate astrocyte and microglial reactivity, focusing on the stratum *radiatum* of the CA1 region, between Bregma −3.14 to −4.16 mm (Paxinos and Watson, 1998) according to our previous studies (Ezquer et al., 2019; Israel et al., 2019) and to Gomez et al. (2018).

Since, as indicated, the prefrontal cortex is an important region in the glutamatergic decision-making system (Kalivas, 2009); while little is known about the effects of nicotine exposure on GLT-1 and xCT expression, we examined the mRNA levels of both GLT-1 and xCT in the prefrontal cortex of rats following the reinstatement of oral consumption of a nicotine solution post-deprivation. The medial prefrontal cortex, including cingulate and pre- and infralimbic regions, between Bregma +3.2 and +1.7 mm (Paxinos and Watson, 1998), was selected for quantification of the mRNA levels of GLT-1 and the xCT cystine/glutamate antiporter.

### Determination of Astrocyte and Microglial Immunoreactivity

Immunofluorescence against the astrocyte marker, glial fibrillary acidic protein (GFAP), and the microglial marker ionized−calcium–binding adaptor molecule 1 (Iba−1) were evaluated in coronal cryo−sections of the hippocampus (30 μm thick) as previously reported (Ezquer et al., 2019). Nuclei were counterstained with DAPI. Microphotographs were taken from the *stratum radiatum* of the hippocampus using a confocal microscope (Olympus FV10i). The area analyzed for each stack was 0.04 mm^2^, and the thickness (*Z* axis) was measured for each case. The total length and thickness of GFAP-positive astrocyte primary processes and the density of Iba−1–positive microglial cells were assessed using FIJI image analysis software^1^ as previously reported (Ezquer et al., 2019).

### Glutathione Determination in the Hippocampus

Brain oxidative stress was determined by assessing the ratio of oxidized (GSSG) to reduced (GSH) glutathione in the hippocampus as previously described (Ezquer et al., 2019). Glutathione reductase (G3664), NADPH (N1630), and DTNB (5,50−dithiobis−2−nitrobenzoic acid), used for the determination of glutathione, were purchased from Sigma-Aldrich.

### Quantification of mRNA Levels of GLT−1 and xCT Cystine/Glutamate Exchanger in the Prefrontal Cortex

Two hours after recording the nicotine relapse (day 128), the animals were anesthetized with chloral hydrate (280 mg/kg, i.p.) and euthanized to obtain prefrontal cortex samples. Total RNA from the prefrontal cortex was purified using Trizol (Invitrogen, Grand Island, NY, United States). One microgram of total RNA was used to perform reverse transcription with MMLV reverse transcriptase (Invitrogen) and oligo dT primers. Real-time PCR reactions were performed to amplify the glutamate transporters GLT1 and xCT using a Light-Cycler 1.5 thermocycler (Roche, Indianapolis, IN, United States). The primers used for qPCR amplifications were designed by the authors using the following sequence: GLT-1 sense 5′-CCTCATGAGGATGCTGA AGA-3′ GLT-1 antisense 5′-TCCAGGAAGGCATCCAGGC TG-3′ and xCT sense 5′-CCTGGCATTTGGACGCTACAT-3′ xCT antisense 5′-TCAGAATTGCTGTGAGCTTGCA-3′. To ensure that amplicons were generated from mRNA and not from genomic DNA, controls without reverse transcriptase during the reverse transcription reaction were included. Relative quantifications were performed by the ΔΔCT method. The mRNA level of each target gene was normalized against the mRNA levels of the housekeeping gene glyceraldehyde-3-phosphate dehydrogenase (GAPDH) in the same sample.

### Induction of Voluntary Oral Consumption of a Nicotine Solution

The voluntary oral consumption of a nicotine solution was induced as was previously described (Quintanilla et al., 2018, 2019). Briefly, twenty-eight female naïve UChB rats were intraperitoneally administered 0.6 mg/kg/day of nicotine hydrogen tartrate (Bashiri et al., 2016), for 14 consecutive days. On day 15, 24 h after nicotine intraperitoneal injections were discontinued, all rats were given continuous (24 h/day) two-bottle free choice access between water and a nicotine solution. The concentration of the nicotine solution, calculated as free base, was 5 mg/L (w/v) on the initial 3 days and was increased to 10 mg/L from days 4 to 6, to 30 mg/L from days 7 to 9, and to 50 mg/L from days 10 to 15. Finally, from day 16 onward, the nicotine concentration was raised to 60 mg/L and was kept constant for 4 months. Oral nicotine and water intakes were recorded daily, and the bottle positions were alternated every day to avoid the development of a side preference. Oral voluntary nicotine intake was expressed as milligrams of nicotine consumed per kilogram body weight per day (mg/kg/day), and water intake was expressed as ml of water consumed per kilogram body weight per day (mL/kg/day).

### Experiment 1A. Effect of the Oral Administration of NAC, ASA, or NAC + ASA on the Oral Voluntary Consumption of a Nicotine Solution

Once a sustained voluntary nicotine intake was achieved, as described above, the 28 female UChB rats were given continuous (24 h/day) free-choice access to a nicotine solution (60 mg/L; w/v) and water, for 112 days. On day 84, rats were randomly assigned to four groups (*n* = 7 rats/group), which received for 11 consecutive days one of the following treatments: (1) Vehicle group: rats were given water, by oral gavage; (2) NAC group: rats were given *N*-acetylcysteine (NAC) (40 mg/kg/day), by oral gavage; (3) ASA group: rats were given ASA (15 mg/kg/day), by oral gavage; and (4) NAC + ASA group: rats were administered the combination of NAC (40 mg/kg/day) plus ASA (15 mg/kg/day), by oral gavage. It is important to note that access to oral nicotine-solution intake was not interrupted during the 11 days while NAC, ASA, or NAC + ASA was administered, nor was it interrupted for the following additional 17 days posttreatment (washout period). Voluntary oral nicotine consumption was recorded daily and expressed as milligrams of voluntary nicotine consumed orally per kilogram body weight per day (mg/kg/day). Water intake was expressed as ml of water consumed per kilogram body weight per day (ml/kg/day).

### Experiment 1B. Effect of Oral Administration of NAC, ASA, or NAC + ASA on the Reinstatement of the Oral Nicotine Consumption Post-deprivation

After 113 days of voluntary chronic oral nicotine intake, the animals of experiment 1A were deprived of nicotine, but not of water, for 14 days, and thereafter allowed re-access to a nicotine solution (60 mg/l; w/v) for 2 days. On the last 9 days of nicotine deprivation, rats were divided into four groups (*n* = 7) that received a daily dose of the following: (1) Vehicle group: rats were administered water, by oral gavage (Conybeare and Leslie, 1988); (2) NAC group: rats were administered *N*-acetylcysteine (40 mg/kg/day) by oral gavage; (3) ASA group: rats were administered ASA (15 mg/kg/day) by oral gavage; and (4) NAC + ASA group: rats were administered a combination of NAC (40 mg/kg/day) plus ASA (15 mg/kg/day) by oral gavage. The last treatment dose was administered 24 h prior to the reinstallation of 60 mg/l (w/v) nicotine solution (on day 126), for two continuous days (days 127 and 128). Once the experiment had ended, after recording the voluntary oral nicotine intakes at the second day (day 128) of nicotine re-access, animals were anesthetized with chloral hydrate (280 mg/kg, i.p.), perfused intracardially with 100 ml of PBS (pH 7.4), and euthanized to obtain brain samples for determination of the GSSG/GSH ratio, to assess astrocyte and microglial immunoreactivity in the hippocampus and for quantification of mRNA levels of the GLT-1 and xCT cystine/glutamate exchanger in the prefrontal cortex. The rat estrous cycle was not monitored since neither the estrous cycle nor sex differences have been reported to influence the reinstatement of extinguished nicotine-seeking behavior in male and female rats (Feltenstein et al., 2012).

### Experiment 2. Effect of the Inhibition of the xCT-Cystine/Glutamate Exchanger on Oral Voluntary Consumption of a Nicotine Solution

To study the participation of the xCT cystine/glutamate antiporter in the inhibitory effect of *N*-acetylcysteine on chronic nicotine intake, we administered sulfasalazine (SZ), a potent inhibitor of this transporter (Gout et al., 2001). For this study, we used a new group of twenty adult female UChB rats, which after being induced to voluntarily consume a nicotine solution orally as described above were given access to nicotine (60 mg/L; w/v) and water for 91 days. On day 83, rats were divided into four groups (*n* = 5 rats/group), which received for two consecutive days the following treatments: (1) Vehicle/vehicle group: rats were administered saline (NaCl 0.9%) via i.p. (5 mL/kg), as was described above, 15 min before water administration (5 ml/kg) by oral gavage, as was described above; (2) sulfasalazine/NAC group: rats were administered sulfasalazine, an inhibitor of xCT-cystine/glutamate exchanger, at a dose of 8 mg/kg/day (i.p.) (Bernabucci et al., 2012), 15 min before NAC administration (40 mg/kg/day) by oral gavage; (3) sulfasalazine/vehicle group: rats were administered SZ at a dose of 8 mg/kg/day via i.p., as was described above, 15 min before water administration (5 ml/kg/day), by oral gavage; and (4) vehicle/NAC group: rats were administered saline (NaCl 0.9%) via i.p. (5 mL/kg) 15 min before the administration of a dose of 40 mg/kg of NAC by oral gavage. Oral voluntary nicotine intake was recorded daily and expressed as milligrams of nicotine consumed per kilogram body weight per day (mg/kg/day).

### Statistical Analyses

Statistical analyses were performed using GraphPad Prism (San Diego, CA, United States). Data are expressed as means ± SEM. The normal distribution of data for all experiments was first tested using the Shapiro–Wilk test. For normally distributed data, one-way analysis of variance (ANOVA) (Figures 2, 4B, 5–7, and 8A) or two-way (treatment × day) ANOVA (Figures 3, 4A, 8B) was used followed by a Tukey or Fisher *post hoc* test. A level of *P* < 0.05 was considered for statistical significance. To facilitate text reading, full statistical ANOVA analyses were presented as shown in the figure legends.

**FIGURE 1:**
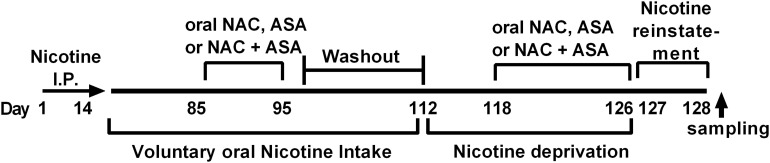
Experimental time-line. The timeline indicates the times at which (i) intraperitoneal nicotine was administered; (ii) free choice of oral nicotine and water was allowed; (iii) NAC, ASA, and NAC + ASA were administered to animals chronically consuming oral nicotine; (iv) nicotine deprivation was carried out; (v) administration of NAC, ASA, and NAC + ASA prior reinstatement of oral nicotine intake and reinstatement; (vi) brain tissue sampling was done.

**FIGURE 2:**
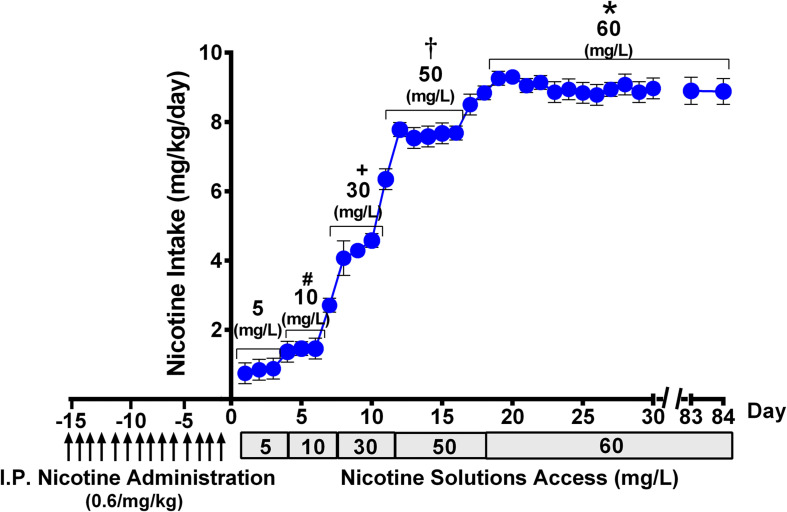
Progressive escalation of voluntary oral nicotine consumption (mean ± SEM) in rats allowed continuous free access to water and a nicotine solution of increasing concentrations, after their pretreatment with an intraperitoneal daily dose of nicotine (0.6 mg/kg), for 14 days. One-way ANOVA of all voluntary oral nicotine intake data indicated a significant effect of the concentration of the nicotine solution offered to the rats [*F_*concentration (4,117)*_* = 977.3, *p* < 0.0001]. Tukey *post hoc* test revealed that when rats were allowed access to nicotine, 60 mg/L displayed higher levels of voluntary oral nicotine intake than when they were allowed access to nicotine 50, 30, 10, or 5 mg/L (*p < 0.0001), and when rats were allowed access to nicotine 50 mg/L displayed higher levels of oral nicotine intake than when they were allowed access to nicotine 30, 10, or 5 mg/L (†*p* < 0.0001). Further, when rats were allowed access to nicotine, 30 mg/L displayed higher levels of oral nicotine intake than when they were allowed access to nicotine 10 or 5 mg/L (+*p* < 0.0001), while when rats were allowed access to nicotine 10 mg/L displayed higher levels of oral nicotine intake than when they were allowed access to nicotine 5 mg/L (#*p* < 0.0001).

**FIGURE 3:**
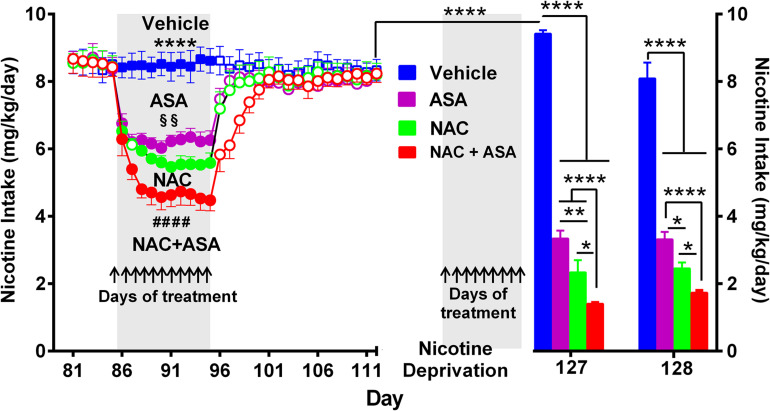
The coadministration of *N*-acetylcysteine (NAC; 40 mg/kg/day p.o.) plus acetylsalicylic acid (ASA; 15 mg/kg/day p.o.) induced a significantly greater inhibition of both chronic oral nicotine intake **(left)** and post-deprivation reinstatement **(right)** in rats that had chronically consumed nicotine. **(Left)** following 12 weeks of chronic voluntary oral consumption of the nicotine solution, animals were treated with either *N*-acetylcysteine, acetylsalicylic acid, *N*-acetylcysteine + acetylsalicylic acid, or vehicle by oral gavage, for 11 consecutive days. Two-way ANOVA (treatment × day) of nicotine intake data revealed a significant effect of treatment [*F*_*treatment*__(3,239)_ = 1839, *p* < 0.0001], day [*F_*day (11,239)*_*_=_ 28.44, *p* < 0.0001], and treatment x × day interaction [*F_*interaction (33,239)*_* = 17.07, *p* < 0.0001]. Tukey *post hoc* analysis indicated that the groups of rats treated *N*-acetylcysteine (NAC; green circles), acetylsalicylic acid (ASA; pink circles), and *N*-acetylcysteine + acetylsalicylic acid (NAC + ASA; red circles) showed reduced voluntary oral nicotine intake versus that of the control group treated with vehicle (Blue squares) (****p < 0.0001). *N*-Acetylcysteine + acetylsalicylic acid treatment induced a significantly greater reduction of chronic voluntary oral nicotine intake compared with those induced by *N*-acetylcysteine and acetylsalicylic acid alone (####*p* < 0.0001 NAC + ASA compared with NAC and ASA group). In addition, *N*-acetylcysteine induced a significantly higher reduction of oral nicotine intake compared with that induced by acetylsalicylic acid (§§*p* < 0.01 NAC compared with ASA group). **(Right)** following 112 days of voluntary oral consumption of the nicotine solution, animals were treated with either *N*-acetylcysteine, acetylsalicylic acid, *N*-acetylcysteine + acetylsalicylic acid, or vehicle by oral gavage, on the last 9 days (arrows) of a 2-week nicotine deprivation period. Bars show daily oral consumption of the nicotine solution during the 2-day of nicotine re-access, which was carried out 24 and 48 h after treatments were discontinued. Student *t-*test revealed that on the first day (127), the reinstatement of oral nicotine intake of the vehicle group that had consumed oral nicotine for 112 days and was vehicle treated during the nicotine deprivation period (blue bar) was significantly higher compared to the oral nicotine intake of the same group prior to the 14-day deprivation period (mean of nicotine intake of the last eleven baseline days) (empty blue squares) (****p < 0.0001), indicating an increased nicotine seeking behavior. Two-way ANOVA (treatment × day) of the oral nicotine intake data, during the first (day 127) and second (day 128) days of nicotine re-access, indicates significant effect of treatment [*F*_*treatment (3,38)*_ = 335.6, *p* < 0.0001] but not of day. Fisher *post hoc* analysis indicated that, compared with the control group that had consumed oral nicotine for 112 days and was vehicle treated during the nicotine deprivation period (blue bar), the groups treated with *N*-acetylcysteine (NAC; green bar), acetylsalicylic acid (ASA; pink bar), or *N*-acetylcysteine + acetylsalicylic acid (NAC + ASA; red bar) showed a significant reduction of the post-deprivation reinstatement of oral consumption of the nicotine solution (****p < 0.0001 vehicle group versus ASA, NAC, and NAC + ASA) on the first and second days of nicotine re-access (days 127 and 128). *N*-Acetylcysteine + acetylsalicylic acid treatment induced a significantly higher reduction of the reinstatement of oral consumption of the nicotine solution than that induced by acetylsalicylic acid or *N*-acetylcysteine on the first day (****p < 0.0001 NAC + ASA versus ASA; *p < 0.05 NAC + ASA versus NAC) and the second day (****p < 0.0001 NAC + ASA versus ASA; *p < 0.05 NAC + ASA versus NAC) of re-access. In addition, *N*-acetylcysteine induced a significant higher reduction of the reinstatement of oral consumption of the nicotine solution than that induced by acetylsalicylic acid (*p < 0.05 NAC versus ASA) on the 2 days of re-access.

**FIGURE 4:**
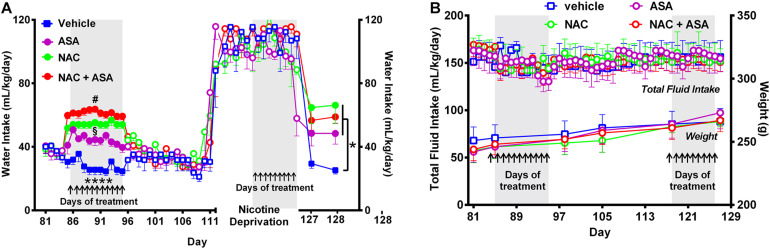
Administration of *N*-acetylcysteine (NAC; 40 mg/kg/day p.o.), acetylsalicylic acid (ASA; 15 mg/kg/day p.o), or *N*-acetylcysteine + acetylsalicylic acid treatment increased oral water intake and did not change fluid intake nor body weight. **(A)** Shows the changes in oral water consumption at the times of chronic voluntary oral nicotine intake, nicotine deprivation, and reinstatement of oral nicotine intake. **(A)** Left, Two-way ANOVA (treatment × day) of oral water intake data following repeated treatment with NAC, ASA, or NAC + ASA or vehicle by oral gavage (day 85–95) at the times of chronic oral nicotine intake revealed significant effect of treatment [*F_*treatment (3,205)* =_* 219.7, *p* < 0.0001], but not of day. Fisher *post hoc* analysis indicated that animals treated with *N*-acetylcysteine (NAC), acetylsalicylic acid (ASA), or *N*-acetylcysteine + acetylsalicylic acid (NAC + ASA) consumed greater amounts of water as compared to that shown by the control group (vehicle treated) (****p < 0.0001 vehicle group versus NAC, ASA or NAC + ASA). Further, *N*-acetylcysteine + acetylsalicylic acid treatment induced a significantly greater increase of oral water intake compared with that induced by *N*-acetylcysteine or acetylsalicylic acid alone (#*p* < 0.05 NAC + ASA versus NAC and ASA), while *N*-acetylcysteine induced a greater increase of water intake compared with that induced by acetylsalicylic acid (§*p* < 0.05 NAC versus ASA). **(A)** Right; following 112 days of chronic free-choice nicotine/water intake, a 14-day nicotine deprivation period followed. On the last 9 days (arrows) of the nicotine derivation period, animals received NAC, ASA, or NAC + ASA. Water was always available throughout the experiment. Two-way ANOVA (treatment × day) performed on water intake data obtained on the first (day 127) and second (day 128) days of nicotine re-access post deprivation revealed a significant effect of treatment [*F_*treatment (3,36)* =_* 7.79, *p* < 0.0004], but not of day. Fisher *post hoc* test indicated that *N*-acetylcysteine (NAC), acetylsalicylic acid (ASA), and *N*-acetylcysteine + acetylsalicylic acid (NAC + ASA) increase oral water intake compared with that of the control group (*p < 0.05). **(B)** The administration of *N*-acetylcysteine, acetylsalicylic acid, or the combination of *N*-acetylcysteine + acetylsalicylic acid did not affect oral total fluid intake [ANOVA: *F*_(3,184)_ = 1.44, *p*: N.S.[, or body weight- [ANOVA: *F*_(3,20)_ = 0.40, *p*: N.S.], compared with vehicle administration.

**FIGURE 5:**
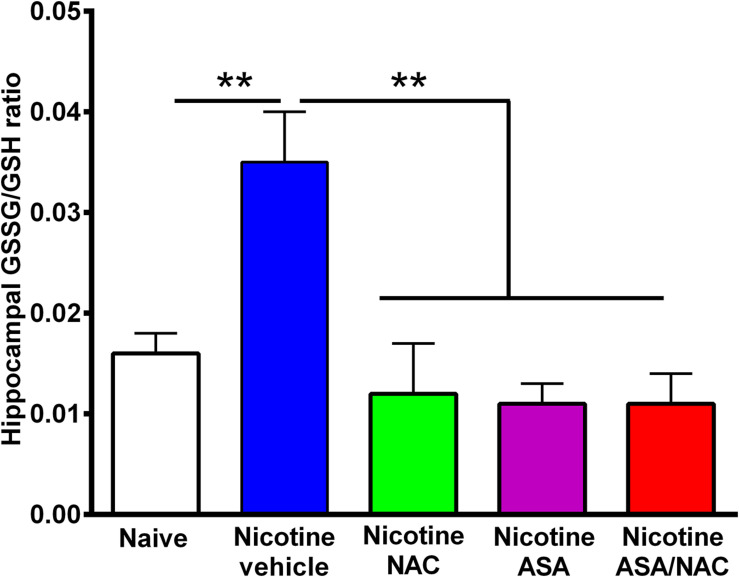
The marked increase in oxidative stress detected in the hippocampus of animals following the post-deprivation reinstatement of oral consumption of nicotine solution was reversed by the administration of *N*-acetylcysteine (NAC; 40 mg/kg), acetylsalicylic acid (ASA; 15 mg/kg/day p.o), or *N*-acetylcysteine + acetylsalicylic acid treatment. One-way ANOVA of all the oxidized/reduced glutathione (GSSG/GSH) ratios data shown indicates a significant effect of treatment [ANOVA: *F*_4,23_ = 9.227, *p* < 0.0001]. Tukey *post hoc* test revealed that the oxidative stress, determined as the ratio of oxidized/reduced glutathione (GSSG/GSH), was markedly increased in the hippocampus of rats following the reinstatement of oral consumption of the nicotine solution and that had been treated with vehicle (blue bar) compared with that of naïve rats drinking only water and were vehicle-treated (white bar) (**p < 0.01). In addition, *N*-acetylcysteine, acetylsalicylic acid, or *N*-acetylcysteine + acetylsalicylic acid fully normalized the nicotine-induced increase in GSSG/GSH ratio [Tukey *post hoc* **p < 0.01 naïve rats drinking only water (white bar) compared with nicotine drinking rats treated with *N*-acetylcysteine (green bar), acetylsalicylic acid (purple bar), or *N*-acetylcysteine + acetylsalicylic acid (red bar)].

**FIGURE 6:**
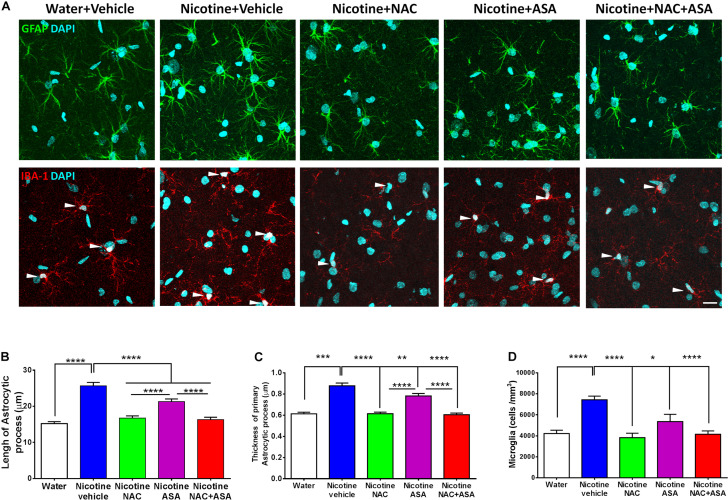
Neuroinflammation detected in the hippocampus of animals following the post-deprivation reinstatement of oral consumption of nicotine solution was inhibited by the administration of *N*-acetylcysteine (NAC; 40 mg/kg), acetylsalicylic acid (ASA; 15 mg/kg/day p.o), or *N*-acetylcysteine + acetylsalicylic acid. **(A)** Representative images of GFAP positive astrocytes (top) and IBA1- positive microglia (center). **(B)** Quantitation of length and **(C)** thickness of GFAP-positive astrocyte primary processes and **(D)** Quantitation of the density of IBA1-positive microglial cells. The top panel shows astrocyte immunofluorescence (GFAP immunoreactivity, green; DAPI, blue). The center panel shows microglia immunofluorescence (IBA-1 immunoreactivity, red; DAPI, blue, depicted by white arrow heads). Scale bar 25 μm. One-way ANOVA of data of primary astrocytic processes of rats in the hippocampus of rats following the reinstatement of oral consumption of the nicotine solution and that had been treated with vehicle (nicotine-vehicle, blue bar) indicates a significant increase of length [*F*_(__4,943)_ = 39.76, **** *p* < 0.0001; *post hoc* ****p < 0.0001], a significant increase of thickness [*F*_(__4,489)_ = 42.10, **** *p* < 0.0001; *post hoc* ***P < 0.001], and a significant increase of microglial density [*F*_(__4,39)_ = 11.14, ****p < 0.0001; Tukey’s *post hoc* ****p < 0.0001] compared with that of naïve rats drinking only water and were vehicle-treated (white bar). The administration of either *N*-acetylcysteine (nicotine + NAC), acetylsalicylic acid (nicotine + ASA), or coadministration of *N*-acetylcysteine plus acetylsalicylic acid (nicotine + NAC + ASA) fully normalized nicotine-induced increase in length (Tukey *post hoc*: NAC, ****p < 0.0001; ASA, ****p < 0.0001; NAC + ASA, ****p < 0.0001); the nicotine-induced increase in thickness (Tukey *post hoc*: NAC, ****p < 0.0001; ASA, **p < 0.01; NAC + ASA, ****p < 0.0001); and the nicotine-induced increase in microglial density (Tukey *post hoc*: NAC, ****p < 0.0001; ASA, *p < 0.05; NAC + ASA, ****p < 0.0001).

**FIGURE 7:**
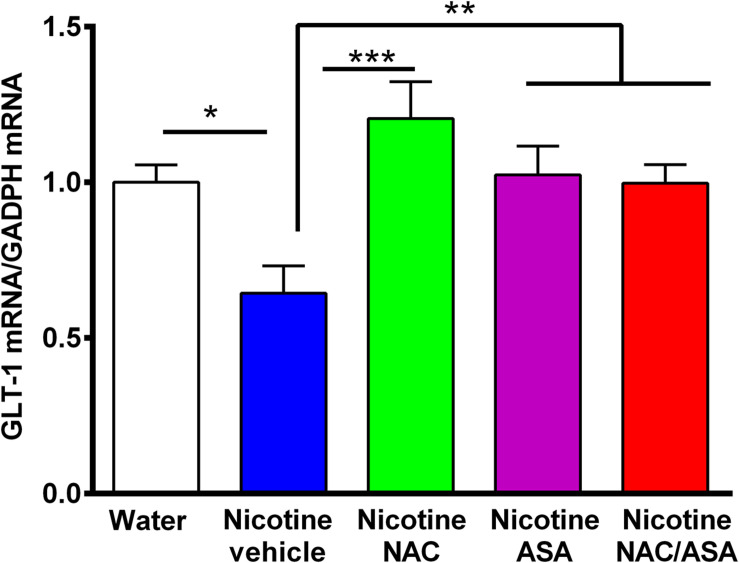
Down-regulation of GLT-1 mRNA detected in the prefrontal cortex of animals following the post-deprivation reinstatement of oral consumption of nicotine solution was reversed by the administration of *N*-acetylcysteine (NAC; 40 mg/kg), acetylsalicylic acid (ASA; 15 mg/kg/day p.o) or *N*-acetylcysteine + acetylsalicylic acid. One-way ANOVA revealed downregulation of GLT-1 mRNA level in the prefrontal cortex of rats following reinstatement of oral consumption of the nicotine solution, and the treatment with vehicle (nicotine-vehicle, blue bar) compared with that of naïve rats drinking only water and were vehicle-treated (naïve, white bar) [One-way ANOVA *F*_(423)_ = 4.961, **p < 0.01; Fisher *post hoc*: *p < 0.05 water group versus nicotine vehicle group, *n* = 7 rats per group], whereas treatment with *N*-acetylcysteine (nicotine-NAC), acetylsalicylic acid (nicotine-ASA), or the combination of both drugs (nicotine-NAC + ASA) normalized GLT-1 mRNA level (***p < 0.001 nicotine-vehicle compared with nicotine-NAC; *P < 0.05 nicotine-vehicle compared with nicotine-ASA and NAC + ASA group).

**FIGURE 8:**
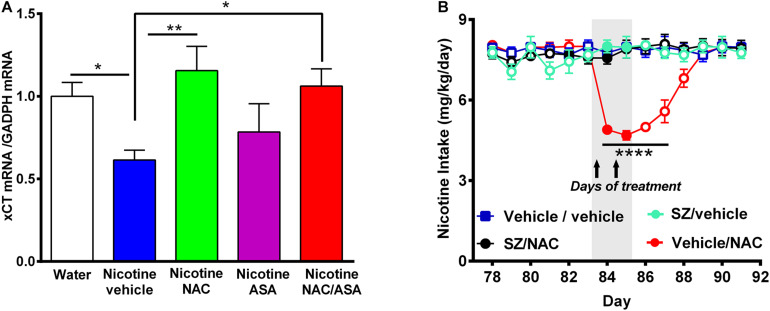
**(A)** Downregulation of xCT-cystine/glutamate exchanger mRNA detected in the prefrontal cortex of animals following the post-deprivation reinstatement of oral consumption of nicotine solution was reversed by the administration of *N*-acetylcysteine or the combination of *N*-acetylcysteine + acetylsalicylic acid. **(B)** In chronically oral nicotine-consuming rats, the inhibition of oral nicotine intake induced by *N*-acetylcysteine (NAC 40 mg/kg) was fully prevented by the xCT inhibitor sulfasalazine (SZ). **(A)** Rats that had shown reinstatement of oral consumption of the nicotine solution and were vehicle-treated (nicotine-vehicle, blue bar) showed downregulation of xCT transporter mRNA level in the prefrontal cortex compared with that of naïve rats drinking only water and were vehicle-treated (naïve, white bar) [One-way ANOVA *F*_(4,19)_ = 3.151, *p < 0.05; Fisher *post hoc*: *p < 0.05 water group versus nicotine vehicle group, *n* = 7 rats per group]. Treatment with *N*-acetylcysteine (nicotine-NAC) or the combination of both drugs (nicotine-NAC + ASA) normalized the expression of the xCT transporter (**p < 0.01 nicotine-vehicle compared with nicotine-NAC, *P < 0.05 nicotine vehicle compared with the nicotine-NAC + ASA group). **(B)** Two-way ANOVA (treatment × day) of all voluntary nicotine oral intake data shown in this Figure revealed a significant effect of *N*-acetylcysteine treatment (vehicle-NAC) [*F*_(3,111)_ = 60.13, ***p < 0.0001], day [*F*_(6,111)_ = 2.622, ***p < 0.02], and also treatment × day interaction [*F*_(18,111)_ = 8.513, ***p < 0.0001]. Fisher *post hoc* indicated that *N*-acetylcysteine treatment induced a marked reduction of the chronic voluntary consumption of a nicotine solution orally from day 84 to 87 (****p < 0.0001 vehicle/vehicle compared with vehicle/NAC), while pretreatment with sulfasalazine, an inhibitor of xCT transporter prevented the reduction of the chronic voluntary consumption of a nicotine solution orally induced by *N*-acetylcysteine.

## Results

Figure 1 shows the experimental timeline indicating the time at which (i) intraperitoneal nicotine was administered; (ii) free choice of oral nicotine and water was allowed; (iii) NAC, ASA, and NAC + ASA were administered to animals chronically consuming oral nicotine; (iv) nicotine deprivation was carried out; and (v) NAC, ASA, and NAC + ASA were administered prior to reinstatement of oral nicotine intake.

Figure 2 shows that following animal pretreatment with an intraperitoneal dose of nicotine (0.6 mg/kg/day) for 14 days, a progressive escalation of oral nicotine consumption was seen in animals that were allowed free choice of water and nicotine solution, the concentration of which was increased every 3–5 days. Following 16 days of oral access to a nicotine solution of 60 mg/L and water, the rats achieved a stable oral nicotine consumption of 8.5 ± 0.1 mg of nicotine/kg of body weight (mean ± SEM). The nicotine concentration of 60 mg/L was kept constant throughout the experiment, excluding the deprivation period. The oral voluntary nicotine intake described in this study was similar to that reported for female rats selectively bred for their high nicotine preference (Nesil et al., 2013) and in naive female rats belonging to the P (high alcohol-preferring) line (Sari et al., 2016). In those studies, animals were given free-choice access of an oral nicotine solution prepared in water adulterated with sucrose, unlike the free-choice access of nicotine dissolved in distilled water that was used in the present work.

Figure 3 (left) shows that the voluntary nicotine intake of control rats (vehicle-treated) that continued to consume nicotine orally following three consecutive months of intake (8.5 ± 0.1 mg/kg/day; mean ± SEM, *n* = 7) was inhibited by 32% by the oral daily administration of *N*-acetylcysteine (NAC, 40 mg/kg/day) (5.8 ± 0.1 mg/kg/day; mean ± SEM; *n* = 7), by 26% by the oral administration of ASA (15 mg/kg/day) (6.3 ± 0.1 mg/kg/day; mean ± SEM, *n* = 7/group), and by 45% by the oral administration of *N*-acetylcysteine + acetylsalicylic acid (4.6 ± 0.1 mg/kg/day; mean ± SEM, *n* = 7), from day 88 to 95. Importantly, the inhibition of oral consumption of the nicotine solution achieved by the combined administration of *N*-acetylcysteine plus ASA was significantly higher than that shown by rats receiving either *N*-acetylcysteine or ASA alone (*p* < 0.0001). Noteworthily, these effects of the antioxidant *N*-acetylcysteine and the anti-inflammatory ASA are seen, while the oral nicotine intake pro-oxidant and pro-inflammatory effects of nicotine continued (*vide infra versus the inhibition on post deprivation reinstatement*). Figure 3 (right) shows the effect on nicotine seeking behavior (relapse/reinstatement) akin to the deprivation followed by the re-access nicotine model (O’Dell and Koob, 2007; Bagdas et al., 2019). Specifically, we assessed the oral consumption of nicotine during the first 24 h of re-access to nicotine (day 127) which followed 14 days of deprivation (blue bar) in rats that had a prior consumption of oral nicotine for 112 days. Water was available throughout the experiment. Results indicate a significant increase of the oral nicotine intake (*p* < 0.0001) in rats of the control group treated with vehicle (9.42 ± 0.1 mg of nicotine/kg/day, *n* = 7; blue bar) compared to the oral nicotine intake of the same group prior to the 14-day deprivation period (8.5 ± 0.1 mg/kg/day; empty blue squares). This increase in oral nicotine intake following 2 weeks of nicotine deprivation referred to as the nicotine deprivation effect (O’Dell and Koob, 2007) reveals a nicotine-seeking behavior in female rats, in line with studies indicating that female rats show a cue-induced nicotine-seeking reinstatement response (Feltenstein et al., 2012; Wang et al., 2014). The increase in the oral voluntary nicotine intake over baseline was temporary, since on the second day of re-access (day 128) the oral nicotine intake of the control group treated with vehicle (8.10 ± 0.5 mg nicotine/kg/day, *n* = 7; blue bar) was not different compared to the nicotine intake of the same group prior to the 14-day deprivation period. To study the effect of the NAC, ASA, and NAC + ASA treatment on the reinstatement of oral consumption of the nicotine solution, the animals were orally administered either (i) *N*-acetylcysteine (40 mg/kg/day), (ii) ASA (15 mg/kg/day), (iii) *N*-acetylcysteine + ASA, or (iv) vehicle, during the last 9 days of the 2-week nicotine deprivation period (indicated by arrows), carried out after 112 days of continuous oral nicotine consumption (see Figure 1). The bars indicate the oral consumption of the nicotine solution on days 127 and 128 (24 and 48 h after discontinuing the NAC, ASA, and NAC + ASA treatments) following the re-access of the nicotine solution (60 mg/l). On day 127 (the first day of re-access of the nicotine solution), the reinstatement of oral consumption of the nicotine solution of the control group (vehicle treated) (9.42 ± 0.1 mg of nicotine/kg/day, *n* = 7; day 127) was inhibited by 75% by *N*-acetylcysteine (NAC) administration (2.3 ± 0.4 mg of nicotine/kg/day; mean ± SEM; *n* = 7; day 127) (*p* < 0.0001) and by 65% by ASA administration (3.5 ± 0.2 mg of nicotine/kg/day; mean ± SEM, *n* = 7; day 127) (*p* < 0.0001). Importantly, the reinstatement of oral consumption of the nicotine solution of the vehicle-treated group was inhibited by 85% by the combined administration of *N*-acetylcysteine plus ASA (NAC + ASA) (1.4 ± 0.04 mg of nicotine/kg/day; mean ± SEM, *n* = 7; red bar) (*p* < 0.0001; day 127). On day 128, the inhibition of the reinstatement of oral consumption of the nicotine solution induced by the administration of NAC, ASA, or NAC + ASA was not significantly different from that obtained on day 127.

Figure 4A shows that NAC, ASA, or NAC + ASA treatment increased the oral consumption of water at the times that they reduced both the oral chronic nicotine intake and the reinstatement of oral consumption of the nicotine solution (Figure 4A, left and right, respectively), such that the total oral intake of fluidics was not modified by the NAC, ASA, or NAC + ASA treatment (Figure 4B). These results, along with the finding that these treatments also did not affect total body weight (Figure 4B), indicate that the changes in oral nicotine consumption by the NAC, ASA, or NAC + ASA treatments do not reflect a non-specific animal malaise.

Figure 5 shows the hippocampal oxidative stress, determined as the ratio of oxidized/reduced glutathione (GSSG/GSH), in rats that had voluntarily consumed nicotine solution orally during 112 days, were deprived of the nicotine solution for 14 days, and were allowed oral nicotine consumption re-access (60 mg nicotine/l) for 2 days. Rats that had shown reinstatement of oral consumption of the nicotine solution and were vehicle-treated (nicotine-vehicle, blue bar) showed a 100% increase (*P* < 0.01) in the GSSG/GSH ratio versus that of control rats that had consumed only water and were water-treated (naïve, white bar). The nicotine-induced oxidative stress was fully normalized by administration of either (a) NAC, (b) ASA, or (c) coadministration of NAC + ASA (*p* < 0.01) during the last 9 days of a 14-day period of nicotine solution deprivation. The marked reduction of the oxidative stress (GSSG/GSH) induced by the anti-inflammatory ASA drug (15 mg/kg/day) is noteworthy.

Figure 6 top, center, and bottom panels show the hippocampal neuroinflammation observed in rats that had voluntarily consumed nicotine solution orally during 112 days, were deprived of the nicotine solution for 14 days, and were allowed oral nicotine consumption re-access (60 mg nicotine/l) for 2 days. Neuroinflammation was seen as an increase in the length (*p* < 0.0001) and thickness (p < 0.001) of the primary astrocytic processes in GAFP-positive cells of rats that had shown reinstatement of oral consumption of the nicotine solution and were vehicle-treated (nicotine-vehicle group, blue bars) versus that of control rats that had consumed only water and were water-treated (naïve rats, white bar) (top and bottom panel A and B). In addition, Figure 6 center panel and bottom panel D indicate that these animals (nicotine-vehicle group, blue bars) also showed a significant increase (*p* < 0.0001) of the density (cells per cubic millimeter) of microglial cells determined by Iba-1 immunoreactivity compared to that of control rats that had consumed only water and were vehicle-treated (naïve rats, white bar). The treatment of the animals during the last 9 days of the 14-day deprivation period of the nicotine solution with either (a) *N*-acetylcysteine (NAC, green bar), (b) acetylsalicylic acid (ASA, pink bar), or (c) coadministration of both drugs (NAC + ASA, red bar) significantly reduced (*p* < 0.05 to *p* < 0.0001) both astrocyte morphology and microglial tissue density—changes most clearly seen in microglial density (*p* < 0.001). The marked anti-inflammatory effect of the antioxidant *N*-acetylcysteine (40 mg/kg/day) on neuroinflammation is noteworthy.

Figure 7 shows that rats that had shown a reinstatement of oral consumption of the nicotine solution and were vehicle-treated (nicotine-vehicle group, blue bars) showed a downregulation of GLT-1 gene expression (mRNA levels) in the prefrontal cortex compared to that of control rats that had consumed only water and were vehicle-treated (naïve rats, white bar) (*p* < 0.05). The treatment of the animals during the last 9 days of the 14-day deprivation period of the nicotine solution with either (a) *N*-acetylcysteine (NAC, green bar), (b) acetylsalicylic acid (ASA, pink bar), or (c) coadministration of both drugs (NAC + ASA, red bar) restored the GLT-1 mRNA level in the prefrontal cortex (*p* < 0.01).

Figure 8A shows that vehicle-treated rats that had voluntarily consumed nicotine solution orally during 112 days, were deprived of the nicotine solution for 14 days, and were allowed oral nicotine consumption re-access (60 mg nicotine/l) for 2 days (nicotine-vehicle, blue bar) showed a downregulation of xCT transporter gene expression (mRNA level) in the prefrontal cortex compared with that of rats drinking only water (naïve group, white bar) (*P* < 0.05). The treatment with *N*-acetylcysteine (nicotine-NAC) or the combination of *N*-acetylcysteine + ASA (nicotine-NAC + ASA) normalized the expression of the xCT transporter gene expression (*P* < 0.05). The treatment with only ASA did not increase the xCT transporter mRNA level. To determine whether the xCT cystine-glutamate transporter plays a role in the mechanism by which *N*-acetylcysteine inhibits nicotine intake, we determined the effect of sulfasalazine (SZ), an inhibitor of the xCT transporter, on the *N*-acetylcysteine-induced reduction of the oral voluntary consumption of a nicotine solution. Results in Figure 8B show that the oral voluntary consumption of a nicotine solution for 83 consecutive days of the control group (vehicle/vehicle group) (7.9 ± 0.1 mg/kg/day; mean ± SEM, *n* = 5) was significantly inhibited by 39.2% (*p* < 0.0001) by the oral administration of NAC (40 mg/kg/day) for a 2-day period (4.8 ± 0.1 mg/kg/day; mean ± SEM; *n* = 5). Pretreatment of animals with sulfasalazine (SZ) (8 mg/kg/day i.p), 15 min before the administration of each of the two *N*-acetylcysteine doses (SZ/NAC group) fully blocked the *N*-acetylcysteine-induced inhibition of chronic voluntary oral consumption of a nicotine solution.

## Discussion

Although rats do not spontaneously ingest nicotine orally, they voluntarily consume nicotine solutions if they became dependent on nicotine following a repeated intraperitoneal administration. The present preclinical study shows that the combined administration of *N*-acetylcysteine (40 mg/kg/day) plus ASA (15 mg/kg/day) reduced by 85% the oral nicotine intake after chronic intake and restatement, a significantly higher inhibition than that generated by each agent alone. The dose of *N*-acetylcysteine (40 mg/kg/day administered orally) used in the present study is within the range used in other studies. An intraperitoneal dose of *N*-acetylcysteine of 30 mg/kg was shown to reduce nicotine self-administration in rats (Ramirez-Nino et al., 2013). The ASA dose used in the present study (15 mg/kg/day) was chosen since doses in the range of 15–30 mg/kg were shown not to generate gastric irritation in rats (Wallace et al., 2004).

In the relapse-like reinstatement condition of oral nicotine intake post-deprivation in rats that had consumed nicotine chronically, the study showed a marked increase in the ratio of oxidized/reduced glutathione (GSSG/GSH) in the hippocampus, an indicator of oxidative stress, which was associated with hippocampal neuroinflammation, as seen both by a greater length and thickness of primary astrocytic processes and by an increase in microglial density. In mice, microglial morphology changes in striatal tissues have been reported following chronic treatment with nicotine and undergoing withdrawn, which were associated with the release of pro-inflammatory cytokines and increase in ROS (Adeluyi et al., 2019; Saravia et al., 2019). Increased TNFα levels have been reported in the nucleus accumbens of rats during cue-induced nicotine seeking (Namba et al., 2020), and an increased TNFα gene expression has also been described in the prefrontal cortex of nicotine- and cigarette smoke-exposed rats (Lau et al., 2012). It is well established that through the activation and nuclear translocation of the transcription factor NF-κB, TNFα induces the synthesis of various pro-inflammatory cytokines, including IL-6 (Shimizu et al., 1990; Kunsch and Rosen, 1993; Ping et al., 1996). The report that the induction of the nuclear transcription of NF-κB by injecting a viral vector expressing an analog of the enzyme IκB kinase (IKK) facilitated cue-induced nicotine seeking reinstatement suggests that the NF-κB pathway and neuroinflammation mediate the conditioned nicotine seeking (Namba et al., 2020).

Two previous sub-chronic nicotine studies did not reveal changes in glutamate transporter-1 (GLT-1) or xCT in the prefrontal cortex of rats receiving nicotine via self-administration or via minipumps for 21 days (Knackstedt et al., 2009) or in mice exposed phasically (not continuous) to e-cigarette vapor containing nicotine (Alasmari et al., 2017). Given the pivotal role of glutamate and the prefrontal cortex in cocaine-seeking behavior, we sought to investigate whether a chronic nicotine intake reduced the mRNA levels of GLT-1 and xCT in the prefrontal cortex of rats that had been subjected to nicotine reinstatement *following 112 days* of continuous nicotine intake (equivalent to many years of nicotine use in a chronic smoker) and a subsequent 2-week deprivation period.

The present study demonstrated that in a reinstatement condition of chronic oral nicotine intake, rats showed a downregulation of both GLT-1 and xCT gene expression in the prefrontal cortex, a region that via the glutamatergic system informs the nucleus accumbens and mediates cue-induced reinstatement and the drug seeking behavior (McFarland et al., 2003; LaLumiere and Kalivas, 2008; Gipson et al., 2013; Smith et al., 2017). Downregulation of GLT-1 and xCT protein expression has been observed in the nucleus accumbens of rats self-administering nicotine while the restoration of GLT-1 expression was associated with a decrease in nicotine self-administration (Knackstedt et al., 2009; Sari et al., 2016). Further, a significant reduction in GLT-1 protein expression has been shown after cue-induced nicotine reinstatement in the nucleus accumbens (Namba et al., 2020). Noteworthily, several studies show that both brain oxidative stress and neuroinflammation downregulates GLT-1. Oxidative stress (ROS) inhibits glutamate transport by the direct inhibition of GLT-1 activity (Trotti et al., 1997, 1998) and also by the formation of adducts of glutamate transporters with lipoperoxidation products like 4-hydroxynonenal and 4-hydroxyhexenal (Schaur et al., 2015), while the pro-inflammatory cytokine TNFα leads to the downregulation of the GLT-1 mRNA level (Szymocha et al., 2000; Wang et al., 2003; Sitcheran et al., 2005). Taken together, these studies support the growing view that conditions present in an oxidative-stress/neuroinflammation self-perpetuating cycle (Berrios-Carcamo et al., 2020) disrupt glutamate homeostasis by impairing glutamate transport in areas involved in the brain reward system, increasing the relapse-promoting effect of drug-related cues, thus sustaining drug craving and subsequent drug consumption (Kalivas, 2009).

Administration of *N*-acetylcysteine combined with an appropriate psychotherapy has been shown to be clinically effective in the treatment of the tobacco use disorder (Prado et al., 2015), while several preclinical studies have shown that *N*-acetylcysteine inhibits cue-induced nicotine reinstatement (Ramirez-Nino et al., 2013; Powell et al., 2019; Goenaga et al., 2020; Moro et al., 2020; Namba et al., 2020). Noteworthily, such an effect of *N*-acetylcysteine was blocked by microinjection of a GLT-1 antisense morpholino into the nucleus accumbens (Namba et al., 2020).

The likely mechanism by which *N*-acetylcysteine reduces oxidative stress was investigated by Zhou et al. (2018) who showed that in rats subjected to traumatic brain injury, the administration of *N*-acetylcysteine amide (NACA), a precursor of *N*-acetylcysteine, attenuated oxidative stress via the activation of the nuclear factor erythroid 2-related factor 2 (Nrf2)-antioxidant response element (ARE) signal pathway. The Nrf2 system activates the transcription of a number of antioxidant enzymes, and additionally, it upregulates the activity of the promoter of the xCT cystine-glutamate transporter gene (Shih et al., 2003; Habib et al., 2015). Thus, *N*-acetylcysteine could reduce the glutamatergic tone *both* by increasing xCT cystine-glutamate antiporter levels and by increasing the levels of cystine as co-substrate for the antiporter, a dual effect leading to the activation of the inhibitory metabotropic mGlu2/3 receptor.

An added xCT co-substrate effect of *N*-acetylcysteine is based on its property of generating both cysteine and cystine (in reducing GSSG into GSH, cysteine becomes oxidized into cystine), thereby increasing the cystine availability for the cystine/glutamate xCT exchanger. Such an added co-substrate effect of the *N*-acetylcysteine enhances the extrusion of glutamate into the extracellular space (Baker et al., 2003) and stimulates the auto-receptor mGlu2/3 negatively, reducing the release of synaptic glutamate (Schoepp et al., 1999). Such a dual effect can explain why *N*-acetylcysteine showed an additive behavioral effect inhibiting the nicotine reinstatement of oral intake over that of ASA but did not show an additive effect on cellular parameters. The relevance of a high activity of the xCT transporter was further shown by the observation that sulfasalazine fully prevented the inhibitory effect of *N*-acetylcysteine on chronic nicotine intake, when administered by itself. This finding is consistent with early studies of Baker et al. (2003) and in line with the fact that the *N*-acetylcysteine metabolite additionally acts on the xCT transporter enhancing glutamate efflux into the extracellular space, likely stimulating the presynaptic mGlu2/3 auto-receptors and negatively modulating synaptic glutamate release (Moussawi and Kalivas, 2010).

The effect of ASA on nicotine intake was associated with a reduction of oxidative stress and neuroinflammation and with an increase in GLT-1 gene expression. A number of studies have shown that ASA activates the synthesis of peroxisome proliferator-activated receptor-gamma (PPAR-γ) (Yiqin et al., 2009; Tang et al., 2014) known to have both strong anti-inflammatory effects (Jiang et al., 1998; Pascual et al., 2007) and also activates brain GLT-1 transcription (Romera et al., 2007), increasing GLT-1 protein levels and glutamate uptake.

Overall, it is suggested that the additive inhibitory effects of ASA and *N*-acetylcysteine on chronic nicotine intake and relapse is due to the fact that both drugs are acting primarily by different mechanisms, suggesting that the coadministration of ASA and *N*-acetylcysteine may be considered for the treatment of nicotine-dependent behaviors.

## Significance

Common elements contributing to the perpetuation of addictive drugs use are (i) cues associated with the setting in which these drugs were used, leading to relapse/reinstatement, mediated from an increased glutamatergic tone which is (ii) associated with drug-induced oxidative stress and neuroinflammation. The present study showed that the coadministration of the antioxidant *N*-acetylcysteine (NAC) plus the anti-inflammatory ASA inhibited by 85% the oral nicotine reinstatement intake seen after a post-deprivation re-access in rats that had chronically consumed a nicotine solution. *N*-acetylcysteine and ASA administration normalized hippocampal oxidative stress and neuroinflammation showing an additive inhibitory effect on oral nicotine reinstatement behavior. Both NAC and ASA activated the synaptic glutamate transporter (GLT-1) gene expression, an effect compounded by an increase in cystine/glutamate xCT transporter gene expression and activity by NAC administration. Thus, acting via different mechanisms, these combined effects are expected to reduce the glutamate tone. Should the present preclinical data be transferable to humans, two well-known available medications may be repurposed as adjuncts in the treatment of nicotine dependent behaviors.

## Data Availability Statement

The raw data supporting the conclusions of this article will be available upon reasonable request.

## Ethics Statement

The animal study was reviewed and approved by Committee for Experiments with Laboratory Animals at the Medical Faculty of the University of Chile (Protocol CBA# 0994 FMUCH). Written informed consent was obtained from the owners for the participation of their animals in this study.

## Author Contributions

MQ: conception and design, collection of data, data analysis, manuscript writing, and final approval of the manuscript. FE: conception and design, collection of data, data analysis, financial support, manuscript writing, and final approval of the manuscript. PM: design, collection of data, data analysis, and final approval of the manuscript. ME: collection of data, data analysis, and final approval of the manuscript. MH-M: conception and final approval of the manuscript. YI: conception and design, financial support, manuscript draft, and final approval of the manuscript. All authors contributed to the article and approved the submitted version.

## Conflict of Interest

The authors declare that the research was conducted in the absence of any commercial or financial relationships that could be construed as a potential conflict of interest.

## Footnotes

1 http://fiji.sc/Fiji
